# Long-Term Outcomes of Patients with Poor Prognostic Factors Following Transanal Endoscopic Microsurgery (TEMS) for Early Rectal Cancer

**DOI:** 10.3390/biomedicines13020521

**Published:** 2025-02-19

**Authors:** Muneeb Ul Haq, Khaled Noureldin, David Mark Pritchard, Arthur Sun Myint, Carrie A. Duckworth, Ngu Wah Than, David M. Hughes, Shakil Ahmed, Muhammad Ahsan Javed

**Affiliations:** 1 Institute of Systems, Molecular and Integrative Biology, The University of Liverpool, Liverpool L69 7BE, UK; jacqueline.dawber@liverpoolft.nhs.uk (D.M.P.); ccf-tr.papillon.clatterbridgecc@nhs.net (A.S.M.); carried@liverpool.ac.uk (C.A.D.); t.ngu-wah1@nhs.net (N.W.T.); ahsanj@liverpool.ac.uk (M.A.J.); 2 The Clatterbridge Cancer Centre NHS Foundation Trust, Liverpool L7 8YA, UK; 3 Liverpool University Hospitals NHS Foundation Trust, Liverpool L7 8YE, UKdmhughes@liverpool.ac.uk (D.M.H.); shakil.ahmed@liverpoolft.nhs.uk (S.A.); 4 Department of Health Data Science, Institute of Population Health, The University of Liverpool, Liverpool L69 3GF, UK

**Keywords:** rectal cancer, adjuvant treatment, local excision

## Abstract

**Background:** Transanal endoscopic microsurgery (TEMS) is an organ-preserving approach for treatment of early rectal cancer (ERC). However, adverse histopathological features identified post-TEMS often necessitate adjuvant therapy. This study aims to compare the long-term oncological outcomes of patients who underwent TEMS and were offered adjuvant treatments with total mesorectal excision (TME), chemoradiotherapy (CRT), radiotherapy (RT), active surveillance, or dose escalation with contact X-ray brachytherapy (CXB). **Methods:** This study included patients treated with TEMS for ERC between September 2012 and December 2022, with follow-up until December 2023. Patients with adverse histopathological features (extra-mural venous invasion, lympho-vascular invasion, R1 margins, tumour budding) were assigned to adjuvant treatments. Inverse probability of treatment weighting (IPTW) was applied to mitigate selection bias. **Results:** Of the 117 patients, 24 underwent TME, 17 received CRT, 25 received RT, 14 underwent active surveillance, and 37 patients received CXB boost along with CRT. The median follow-up was 60 months (IQR 52–73). During this time, 29 patients developed recurrence, and 15 died. The 5-year overall survival (OS) was 78.6%, and disease-free survival (DFS) was 70.9%. Compared to CXB, the mortality risk for CRT (HR = 0.81; 95% CI: 0.20–3.28; *p* = 0.77) and TME (HR = 3.68; 95% CI: 0.46–29.79; *p* = 0.22) was not significantly different. However, TME was associated with a significantly higher recurrence risk compared to CXB (HR = 7.57; 95% CI: 1.23–46.84; *p* = 0.029). **Conclusions:** An organ-preserving strategy with CRT or CRT combined with a CXB boost may offer comparable long-term outcomes and reduced recurrence risks for patients undergoing TEMS for ERC with poor prognostic features. Further research with larger cohorts is needed to validate these results.

## 1. Introduction

Colorectal cancers rank as one of the most prevalent malignancies in the world, with rectal cancers constituting nearly one in every three newly diagnosed colorectal cancer cases [1]. With the extended age cohort of bowel cancer screening in countries such as the United Kingdom, the detection of rectal cancers at earlier stages and in younger patients is expected to significantly increase in the coming years [2].

Although surgery in the form of total mesorectal excision (TME) of the rectum remains the gold standard treatment for the majority of rectal cancers, those patients who have early rectal cancer may be able to benefit from an organ-sparing approach utilising local excision techniques [3]. According to the European Association for Endoscopic Surgery (EAES) consensus, early rectal cancers are defined as “rectal cancer with good prognostic features that might be safely removed preserving the rectum and that will have a very limited risk of relapse after local excision” [4].

Among these procedures, transanal endoscopic microsurgery (TEMS) increases the quality of local excision and avoids the morbidity associated with other more invasive surgical techniques [5].

The preoperative staging of tumours to assess whether they are suitable for TEMS involves endorectal ultrasound (EUS) along with magnetic resonance imaging (MRI) and computed tomography (CT). This study aims to identify the extent of bowel involvement and nodal involvement that cannot be treated through local resection techniques such as TEMS [6].

However, in a minority of patients who are treated with TEMS, histopathological analysis of the resected specimen identifies a more advanced tumour than was predicted by preoperative imaging assessment. Therefore, additional treatment may be required if there are adverse histopathological features. Options for further management in this situation include completion total mesorectal excision (TME), postoperative adjuvant radiotherapy (RT), chemoradiotherapy (CRT), and active surveillance [6,7].

More recently, contact X-ray brachytherapy (CXB) has also been employed as adjuvant treatment along with chemoradiotherapy to increase local control after (incomplete) local rectal cancer excision [8]. CXB is a highly targeted therapy, which can deliver a low-energy, high-dose radiation to the target area without collateral damage to the surrounding normal mucosa [9]. In a multicentre large cohort of 194 patients, the Contem1 study reported 6-year local recurrence-free survival and overall survival rates of 91% and 81%, respectively, with a median follow-up of 77 months [10]. These results are comparable to the response rates observed following TME and CRT.

Several poor prognostic features have been identified that predispose patients to having an increase in the likelihood of local recurrence following TEMS. These include involvement of the circumferential resection margin (CRM), resection margins (RM) of less than 1 mm, lymphovascular invasion, and tumour budding [11]. However, there are limited data available on the prognosis of patients who have such unfavourable factors after post-TEMS histology and who then undergo adjuvant treatment [11,12].

Although the response rates of close to 80% following CRT, TME, and CXB boost after TEMS are excellent, it is necessary to examine these results in the context of patient-specific factors. This approach will allow for the selection of a regimen that is tailored to the patient’s preferences and risk tolerance (for overall survival and recurrence, which necessitates salvage surgery) [11].

Additionally, there is a need to compare curative treatment options after adjusting for covariates. This will help provide clinical evidence for the efficacy of CRT in terms of overall and disease-free survival outcomes when compared to salvage with TME. This will help patients decide between operative and non-operative management.

It will also provide context to any potential clinical benefits of dose escalation with a CXB boost compared to CRT alone [9]. In addition, it will compare the survival outcomes in this novel group to patients treated with TME.

Therefore, this study aims to report on the long-term outcomes in rectal cancer patients who had adverse histopathological features following TEMS and who subsequently underwent one of five different treatment options (CRT, TME, CXB, RT, and active surveillance).

## 2. Materials and Methods

### 2.1. Study Design

This paper builds on a previous study by Javed et al., which evaluated the outcomes of a cohort of rectal cancer patients who underwent adjuvant treatment by TME, CRT, RT, or active surveillance after TEMS [13]. Following the protocol of this previous study, the recruitment period was extended to cover the period from 1 September 2012 to December 2022, with the last follow-up being on 31 December 2023.

Building on the prospectively maintained database of ERC patients at our institution, new patients were identified at the time of discussion at the regional specialist early cancer multidisciplinary team meeting (SERC-MDT).

However, due to historically low numbers of referrals for CXB within our SERC-MDT cohort, in this study, an additional group of patients was also included who had been treated with CXB boost as adjuvant treatment following TEMS. These patients were identified from a prospectively maintained institutional database from the Clatterbridge Cancer Center. This tertiary care centre receives referrals from clinicians across the United Kingdom (including patients discussed in the SERC-MDT) and historically was one of the first centres to start offering CXB to patients. All patients in the CXB boost arm were referred for treatment after discussion at a local Clatterbridge Cancer Center MDT meeting between 2003 and 2020.

### 2.2. Inclusion and Exclusion Criteria

All patients included in this study underwent preoperative assessment, which confirmed primary rectal adenocarcinoma, which was ≤3 cm, staged as T1, or intermediate between T1 and T2. This study only included patients who presented without lymph node involvement (N0) or distant metastases (M0). Additionally, the depth of invasion was confirmed for all patients using preoperative MRI and/or endorectal ultrasound.

This selection criteria for patients with early rectal cancers who were offered TEMS ensured a low likelihood of mesorectal disease, thereby minimising recurrence risk while preserving sphincter function.

Patients who received neoadjuvant treatment or palliative contact X-ray brachytherapy following local surgery or who had benign rectal tumours or rectal neuroendocrine tumours were excluded from this study.

Patients included in the CXB arm received a standard 60 Gy CXB boost regimen, delivered in two fractions (30 Gy each) over two weeks using a stainless rigid applicator sized at 2.2, 2.5, or 3 cm in diameter.

In the CXB boost arm, only patients who had received the same long-course radiotherapy dose–time fractionation schedule as was outlined in the original study by Javed et al. [13] were included.

### 2.3. Investigations

Investigations used for preoperative staging comprised a pelvic MRI scan and endorectal ultrasound (EUS) performed by one of two dedicated gastroenterologists. A computed tomographic (CT) scan of the chest, abdomen, and pelvis was performed to rule out distant metastases.

### 2.4. Data Collection

Patient details, including age, gender, body mass index (BMI), and American Society of Anesthesiologists (ASA) score, were collected. Additionally, rectal lesion characteristics such as tumour (T) stage on MRI and EUS, post-operative histology, and TME quality based on the magnetic resonance imaging and Rectal Cancer European Equivalence Study criteria (MERCURY) criteria (in cases that underwent surgical resection) were documented [14].

Adverse features identified on post-TEMS histology that prompted offering adjuvant therapy included lymphovascular/perineural invasion, tumour budding, poorly differentiated tumours, or an R1 resection margin. Other concerning features included pedunculated polyps scored as Haggitt level 3 and above or sessile polyps graded as Kikuchi SM3.

### 2.5. Follow-Up of Study Participants

Any tumour development at or near the site of the TEMS resection that developed more than six months following the procedure was deemed a recurrence (after confirmation by biopsy). MRI was used to evaluate regional recurrence, and CT scans, or 18F-FDG PET/CT scans, were used to assess for distant metastases. All findings were discussed at MDT meetings before defining whether they were extra-mucosal recurrences.

Additionally, a flexible sigmoidoscopy was performed biannually, with carcinoembryonic antigen testing, MRI of the rectum, and a CT scan conducted annually during the first two years. For the following three years, surveillance continued according to the UK post-resection guidelines for colorectal cancers [15].

### 2.6. Statistical Methods

Categorical variables were expressed as counts and percentages, while continuous variables were presented as medians with interquartile ranges (IQR). The primary outcomes of interest, disease-free survival (DFS) and overall survival (OS), were compared using Kaplan–Meier survival curves and the log-rank test for significance.

Propensity scores were estimated using a logistic regression model, including covariates such as tumour stage, size, invasion status, age, and R1 status. Nearest neighbour matching with a calliper of 0.25 standard deviations was employed to match treatment groups.

Inverse probability of treatment weighting (IPTW) was utilised to mitigate selection bias and strengthen causal inference. Propensity scores were used to weight subjects, creating a pseudo-population where the distribution of covariates is independent of treatment assignment.

Weighted Kaplan–Meier curves and Cox proportional hazards regression models were used to estimate treatment effects. These models were adjusted for potential confounders, providing hazard ratios (HR) with 95% confidence intervals (CI). A time-dependent Cox model was utilised in situations where the proportional hazards (PH) assumption was violated, such as when Kaplan–Meier curves intersected.

Covariate balance between treatment groups was assessed using standardised mean differences (SMD) both before and after IPTW. An SMD of between 0.1 and 0.2 indicated adequate balance between groups.

The statistical analyses were conducted using RStudio Team (2023), version 2023.06.1+524. RStudio: Integrated Development for R. RStudio, PBC, Boston, MA. URL http://www.rstudio.com/ [16].

## 3. Results

**Figure A1:**
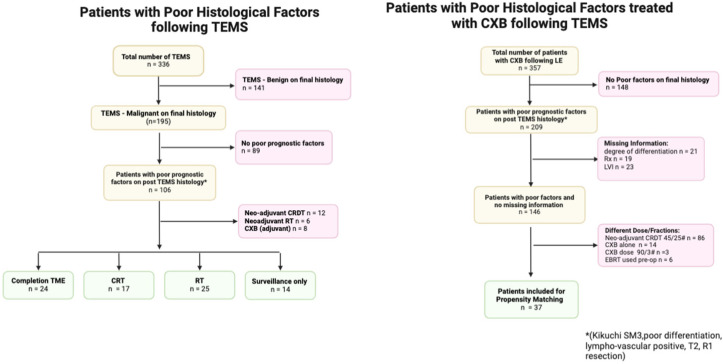
Patient recruitment process for this study.

A total of 117 patients were recruited. This includes an additional 27 patients to the original cohort of 53 patients that we previously reported on and a separate group of 37 patients who had received CXB boost in addition to CRT as adjuvant treatment following TEMS. The recruitment process for this augmented cohort is represented in Figure A1.

The median age of patients was 74.5 (IQR: 64–82.5) years, more patients were male (63.2%), and the median BMI of this study cohort was 25.9 kg/m^2^ (IQR: 23.65–29.85). The median diameter of rectal tumours (on pre-excision scans) was 31 mm (IQR: 20–40), and the median distance from the anal verge was 6 cm (IQR: 2–16 cm).

High-risk features identified following TEMS included poorly differentiated tumours (6.8%) and tumour budding (10%). One in five patients (22.2%) had R1 resection margins, and the same proportion demonstrated lymphovascular invasion (23.1%). More than half of the tumours (50.4%) had submucosal involvement, and just less than half of all tumours (46.2%) were staged as T1 tumours. This is shown in Table 1. Patients were assigned adjuvant treatment based on MDT discussions and patient preferences.

Within this augmented cohort, most patients underwent radiotherapy RT (n = 25) or completion TME (n = 24). Chemoradiotherapy (CRT) was administered in 17 patients, while 14 patients had active surveillance only. Although the newly recruited patients were significantly older than the previous cohort (*p* < 0.01), the two groups otherwise demonstrated similar patient demographics, tumour characteristics, and poor prognostic factors (Table 1).

Notably, the surveillance group included one patient who declined surgery due to frailty. Moreover, 5 patients who were initially offered RT/CRT underwent subsequent surgery, and 2 cases underwent salvage procedures following local recurrence during surveillance.

Similarly, in the CXB boost arm, two patients underwent abdominoperineal resection (APR), and three patients needed palliative radiotherapy for recurrences identified following adjuvant treatment with CXB boost.

**Figure 1:**
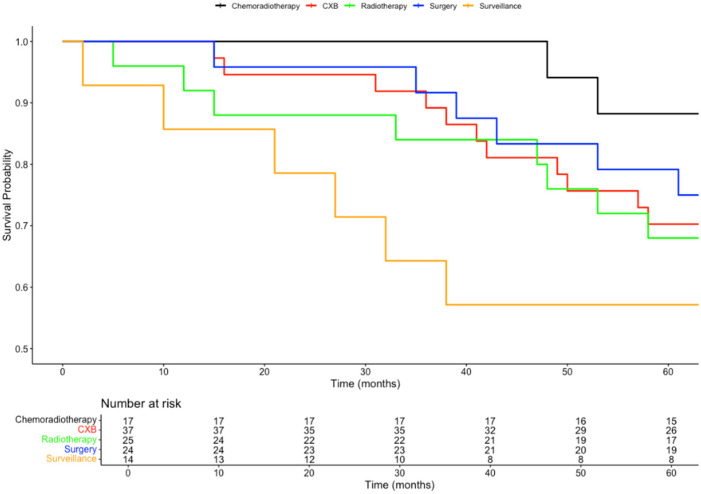
Kaplan–Meier plot comparing the overall survival probabilities of all adjuvant treatment groups.

Patients who received CRT had a 5-year overall survival (OS) rate of 88%, compared to nearly 80% OS for those undergoing TME surgery. In comparison, the 5-year OS rates were similar for patients receiving RT alone (68%) and patients receiving dose escalation with CXB after CRT (70%). The OS at 5 years for patients who opted for no further treatment was 57%. The differences in overall survival rates among these groups were statistically significant (*p* = 0.03), as shown in Figure 1.

**Figure 2:**
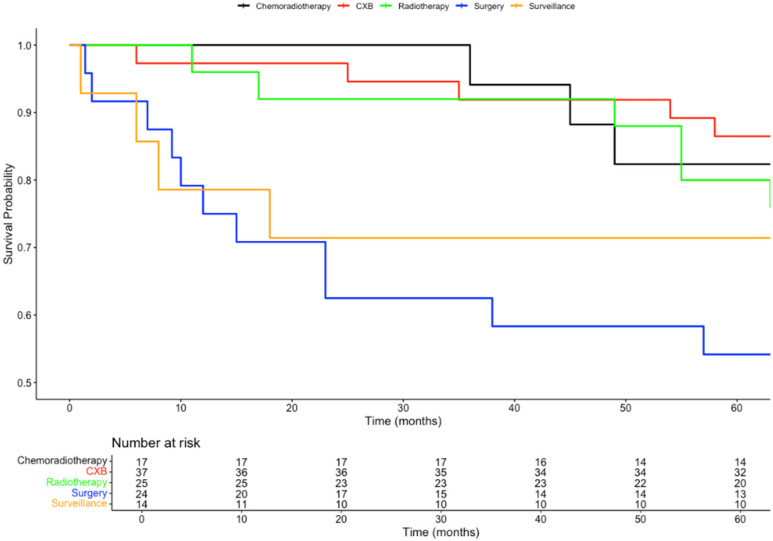
Kaplan–Meier survival plot comparing the disease-free survival probabilities of all adjuvant treatment groups.

Patients receiving a CXB boost in addition to CRT experienced an improved 5-year disease-free survival (DFS), increasing from 82% for CRT alone to 86%. In contrast, patients receiving RT alone had a 5-year DFS of 80%, and those opting for no further treatment had a 5-year DFS of 71%. Patients treated with TME surgery alone had the lowest 5-year DFS, at 54%. The differences in recurrence rates among these groups were also statistically significant (*p* = 0.03). This is represented in Figure 2. The survival outcomes of patients assigned each treatment option are summarised in Table 2.

Table 3 shows the baseline differences between the three curative treatment arms as follows: TME, CRT, and CXB boost. All the groups had similar proportions of patients who had R1 resection margins, lymphovascular invasion, and tumours smaller than or equal to 3 cm. Although a greater proportion of T2 and above tumours were offered adjuvant treatment with TME (66.7%) compared to CXB (43.2%) and CRT (35.3%), this trend was not statistically significant (*p* = 0.09).

Patients who were offered a CXB boost had a median age of 80 years (IQR: 72–84), which was significantly older than patients who were offered CRT (*p* = 0.01) and the TME arm (*p* = 0.001).

### 3.1. Balance of Covariates Before and After Matching

Due to the limited sample size of individual treatment arms, propensity score matching (PSM) led to worsened balance for most of the covariates compared to baseline. For instance, the imbalance in tumour size increased significantly with standardised mean difference (SMD) exceeding 1 for comparisons between CRT and CXB and CRT and TME. Similarly, the balance of patients’ age deteriorated, with an SMD of 1.6 observed in the comparison between CRT and CXB arms.

By contrast, IPTW was better suited for our dataset. It demonstrated a small imbalance (SMD < 0.2) for the comparison between TME and CXB for all variables except age, which had moderate imbalance (SMD > 0.5).

After matching patients treated with CRT and TME with IPTW, all covariates had SMD less than 0.5, with only the stage of the tumour and patients’ age demonstrating an imbalance between groups (SMD > 0.5).

However, for comparisons between CRT and CXB boost, most variables had moderate imbalance (0.2 ≤ SMD < 0.5), except age, which had an SMD of 1.

**Figure A2:**
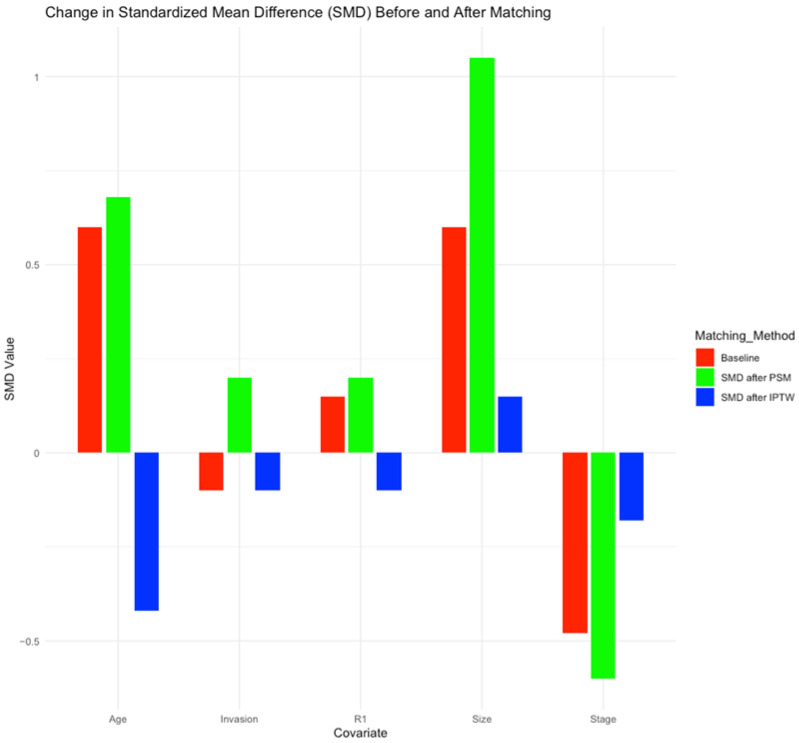
This bar chart shows the changes in standardised mean differences (SMD) for key covariates be-fore and after matching TME vs. CXB.

**Figure A3:**
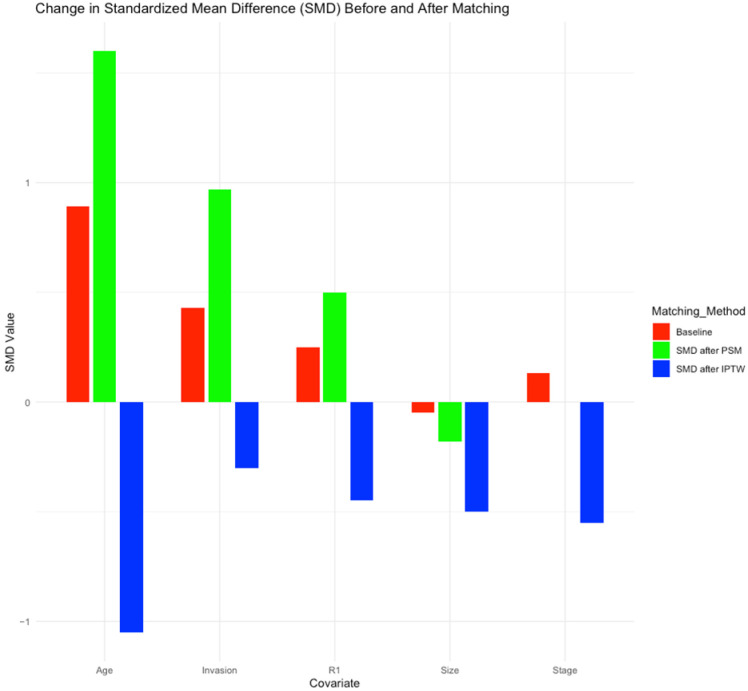
This bar chart shows the changes in standardised mean differences (SMD) for key covariates be-fore and after matching CRT vs. CXB Arm.

**Figure A4:**
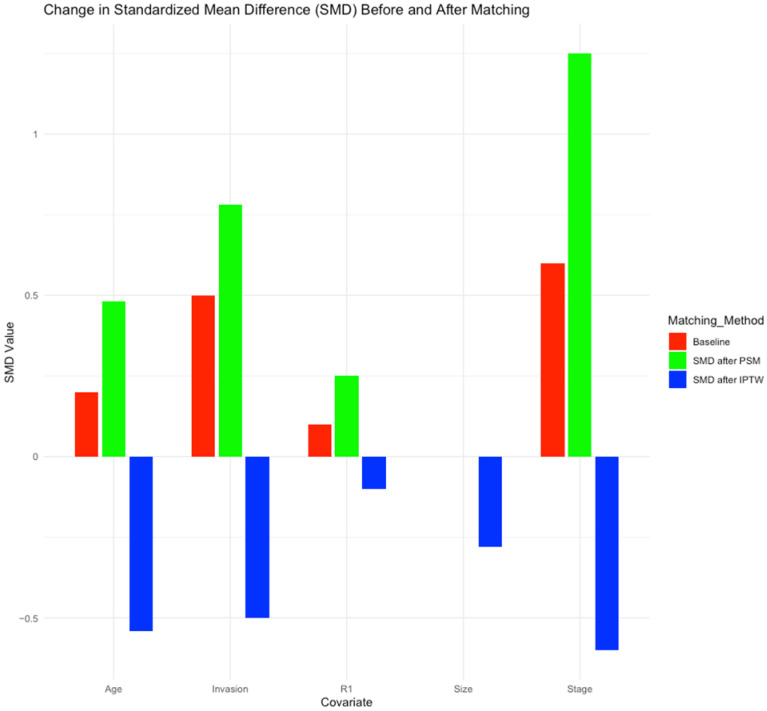
This bar chart shows the changes in standardised mean differences (SMD) for key covariates before and after matching CRT vs. TME Arm.

The balance of covariates between treatment groups at baseline, after PSM, and inverse probability of treatment weighting (IPTW) is illustrated in Figure A2, Figure A3 and Figure A4.

### 3.2. Survival Analysis Between Curative Treatment Outcomes

In a subgroup analysis comparing the three curative treatment options, no significant difference in overall survival (OS) was observed among patients who received CXB, CRT, or TME surgery as adjuvant treatments following TEMS. This trend was consistent for both 3-year OS (89%, 100%, 92%; *p* = 0.4) and 5-year OS (70%, 88%, 80%; *p* = 0.3).

However, patients who received either CXB or CRT experienced significantly higher disease-free survival (DFS) compared to those who underwent completion surgery. This difference was observed at both 3 years (92%, 94%, and 63%; *p* < 0.001) and 5 years (86%, 82%, and 54%; *p* = 0.002).

Given the intersection of Kaplan–Meier survival curves, a time-dependent Cox regression model was used to further analyse survival outcomes among the treatment groups. Compared to CXB, CRT showed a slight reduction in mortality risk (HR = 0.81; 95% CI: 0.20–3.28; *p* = 0.77), while surgery was associated with a higher hazard for mortality (HR = 3.68; 95% CI: 0.46–29.79; *p* = 0.22). However, neither of these findings was statistically significant. For recurrence risk, CRT (HR = 1.69; 95% CI: 0.28–10.28; *p* = 0.57) and surgery (HR = 7.57; 95% CI: 1.23–46.84; *p* = 0.029) both demonstrated increased hazards compared to CXB, with only the elevated risk in the surgery group reaching statistical significance.

To control for baseline differences in confounding variables, including age, gender, tumour size, stage, invasion, and tumour budding, time-dependent Cox models were adjusted using inverse probability of treatment weighting (IPTW). After IPTW adjustment, the differences in 3 year and 5 year OS remained non-significant (*p* = 0.5 and *p* = 0.7, respectively). Similarly, after adjusting for confounders, there were no significant differences in DFS between the three treatment arms at both 3 years (*p* = 0.06) and 5 years (*p* = 0.2).

After IPTW adjustment, the differences in mortality risk remained non-significant for CRT (HR = 0.81; 95% CI: 0.20–3.28; *p* = 0.77) and surgery (HR = 3.68; 95% CI: 0.46–29.79; *p* = 0.22) when compared to CXB.

**Figure 3:**
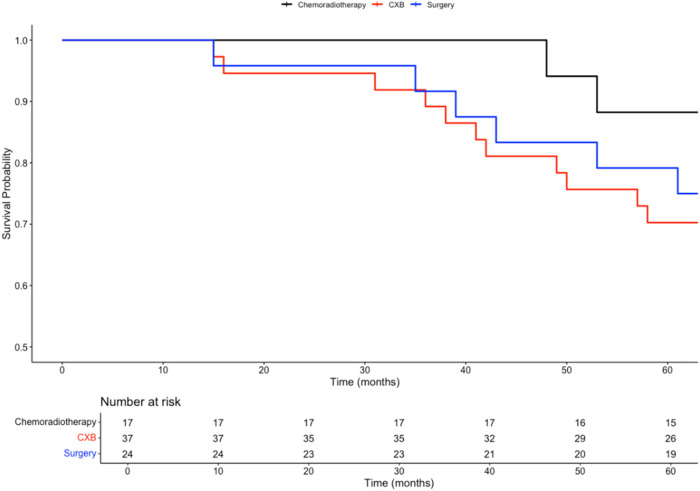
Kaplan–Meier plot comparing the overall survival probabilities of three subgroups: chemoradiotherapy, CXB, and surgery.

**Figure 4:**
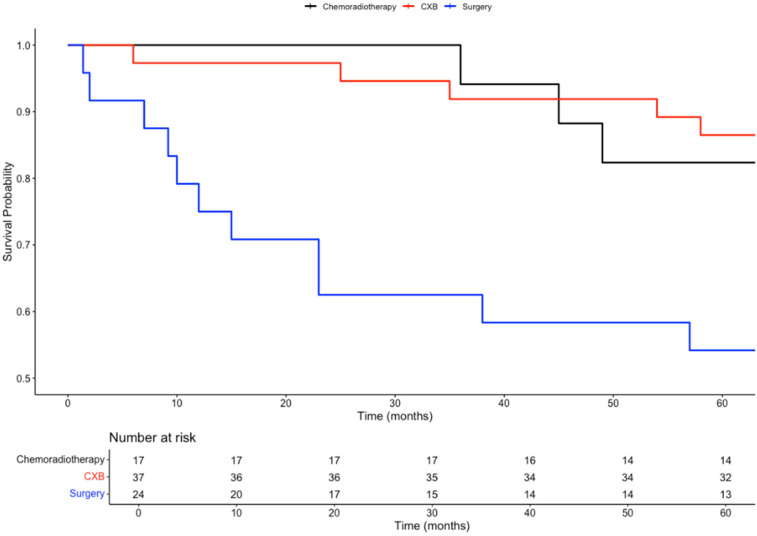
Kaplan–Meier plot comparing the disease-free survival probabilities of three subgroups: chemoradiotherapy, CXB, and surgery.

**Figure 5:**
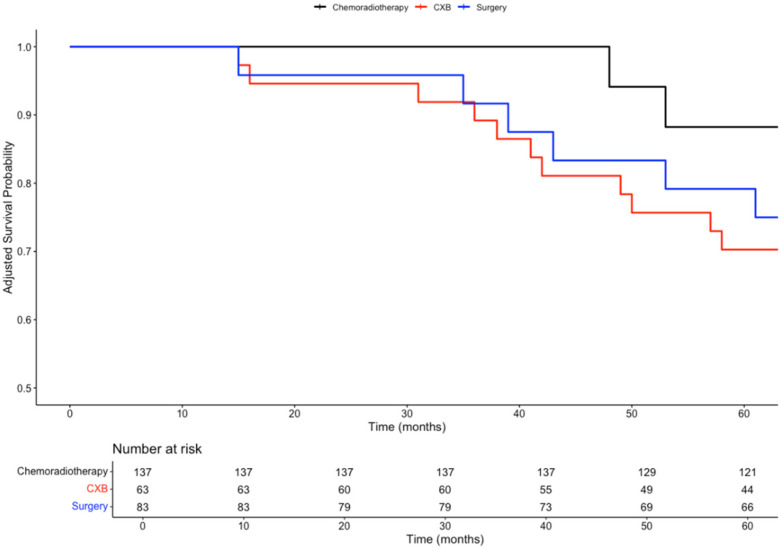
Inverse probability of treatment weighting (IPTW) weighted Kaplan–Meier plot comparing the overall survival probabilities of three subgroups: chemoradiotherapy, CXB, and surgery.

**Figure 6:**
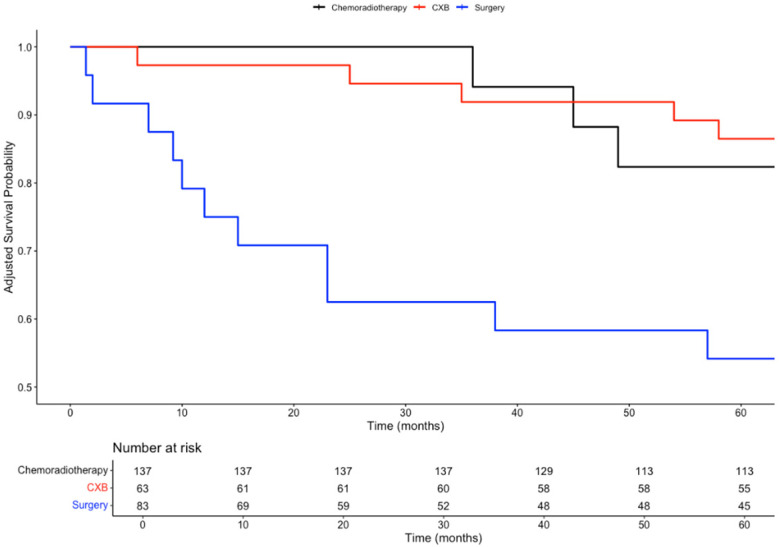
Inverse probability of treatment weighting (IPTW) weighted Kaplan–Meier plot comparing the disease-free survival probabilities of three subgroups: chemoradiotherapy, CXB, and surgery.

For recurrence risk, the IPTW-adjusted analysis showed no significant difference between CRT and CXB (HR = 1.69; 95% CI: 0.28–10.28; *p* = 0.57). However, surgery was associated with a significantly higher risk of recurrence compared to CXB (HR = 7.57; 95% CI: 1.23–46.84; *p* = 0.029), consistent with the findings from the baseline analysis. The survival curves for these comparisons are presented in Figure 3, Figure 4, Figure 5 and Figure 6.

## 4. Discussion

For early rectal cancers, local excision offers a minimally invasive treatment option, which has relatively low morbidity and the potential for functional preservation. However, predicting recurrence following local excision remains challenging, as poor histological factors do not reliably predict recurrence, with lymphovascular invasion being only marginally predictive [17].

Studies have shown that after identification of poor prognostic features on histology, there is a higher rate of local recurrence for patients who do not receive any adjuvant treatment compared to patients who receive completion surgery or CRT [18,19,20].

Given these findings, most patients in our cohort were offered CRT or surgery, with active surveillance and RT alone being reserved for those patients who chose not to undergo more intensive treatment or who were not fit enough for these treatments.

Although, in our previous SERC-MDT case series, no recurrences or deaths were observed in the CRT group [13], the extended follow-up period in this study enabled the capture of some tumour recurrences. Our increased sample size has also reduced the risk of sampling bias relative to the previous publication.

Our unadjusted results report a lower five-year disease-free survival for patients who were offered CRT (nearly 80%) than has been demonstrated in similar studies, which have examined survival in patients who have undergone CRT after local excision of high-risk T1 and T2 tumours. One potential explanation for this trend could be the exclusion of poorly differentiated tumours from some previous studies [5,21,22].

Radical surgery remains the standard of care for rectal cancer, aiming to achieve a clear resection margin [23]. However, our results show a higher recurrence rate in patients who underwent TME compared to CRT. This discrepancy is attributed to seven out of sixteen TME histology reports revealing poor-quality TME (grade 3 TME) according to the magnetic resonance imaging and Rectal Cancer European Equivalence Study criteria (MERCURY) criteria. This was either because of a small bulk of the mesorectum, defects that extend to the muscularis propria, an irregular CRM, or a coning effect of the surgical specimen [14].

Patients undergoing full-thickness TEMS excision are more likely to yield an inferior TME specimen [24]. This is due to fibrotic scarring, which hampers dissection of the correct planes of excision down to the pelvic floor, posing technical challenges [25]. Additionally, standardisation could not be ensured between completion surgery groups, as salvage surgeries were conducted at referring centres, which potentially had varying levels of expertise.

In our analysis, after adjusting for covariates through IPTW, there was no evidence to suggest that there was a statistically significant difference in the overall survival rates between patients who had been treated with CXB boost, CRT, and TME surgery. This may be attributed to the significantly older age of patients in the CXB cohort, which resulted in a substantial imbalance after IPTW matching (SMD > 0.5).

Although dose escalation with CXB demonstrated a lower risk of recurrence after matching with IPTW, this association was not consistently significant on both the Wald test and likelihood ratio test. This might be due to the relatively small sample size of patients in each treatment arm.

### 4.1. Strengths

This study builds upon our previous publication by Ahsan et al. [13] by extending the follow-up period and recruiting additional patients. These additions provide data on the long-term outcomes of patients as well as help minimise the effects of potential sampling bias by increasing the sample size in this study.

Furthermore, this study is novel in its approach of comparing the efficacies of TME, CRT, and dose escalation with CXB boost. In our analysis, following adjustment for patient and tumour-related factors, we present a more accurate comparison between the overall survival and disease-free survival rates between treatment arms.

### 4.2. Limitations

Although this study is highly relevant to the clinical scenario experienced by some patients who have been treated with TEMS for early rectal cancer, its results should be interpreted in the context of several key limitations. The occurrence of poor histological features identified on histology in patients who were initially treated with TEMS is relatively rare. This limited the sample size that our study achieved, and this therefore affected the statistical power of the comparisons made between adjuvant treatment groups. Patients were assigned to adjuvant treatment groups based on MDT discussions rather than randomisation. This may have introduced selection bias into our study.

Additionally, patients who received CXB boost were recruited from a different centre and were recruited over a longer period of time. Despite adhering to the same inclusion and exclusion criteria and monitoring protocols, epidemiological or environmental differences between patients recruited from different centres might influence the generalisability of our results.

Furthermore, variations in data collection both between centres and within individual centers over time meant that certain potentially relevant clinical information, such as the Eastern Cooperative Oncology Group Performance Status, was not consistently recorded.

Future research should focus on patient-reported outcomes, including quality of life and treatment-related adverse events after adjuvant treatment following TEMS. Qualitative studies in this area will also provide valuable insights to help guide clinical decision-making and help tailor the management to the patients’ preferences.

## 5. Conclusions

This multicentre study evaluated the long-term survival outcomes of patients who received adjuvant treatment after TEMS, comparing chemoradiotherapy (CRT), total mesorectal excision (TME), contact X-ray brachytherapy (CXB), radiotherapy (RT), and active surveillance. After adjusting for covariates with IPTW matching, no significant differences were found in the overall survival between patients treated with CXB boost, CRT, and TME. Although CXB boost showed up to a 60% reduction in recurrence risk compared to CRT and TME, results varied across statistical tests, potentially due to small sample sizes. Further research with larger cohorts is needed to provide conclusive evidence about the relative efficacies of these treatment options.

## Figures and Tables

**Table 1:** Patient characteristics.

Variables	New Patients (n = 27)	Original Cohort (n = 53)	New vs. Original Cohort (*p*-Value)	CXB Cohort (n = 37)	Total
Patient Characteristics					
Age (median, IQR)	76 (70–87)	71 (66–78)	0.0056	80 (72–84)	75 (69–82)
BMI, median (IQR), kg/m^2^	27 (25–30)	27.5 (24–30)	0.1782	27 (25 –29)	27 (25–30)
Sex, n (%)					
Male n (%)	20 (74)	39 (74)	1	24 (64.9)	83 (70.9)
Female n (%)	7 (26)	14 (26)		13 (35.1)	34 (29.1)
Lesion Characteristics					
Distance from anal verge, median (range), cm	6 (4.5–8.3)	6 (4.6–8.8)	0.0774	6 (5–7)	3.7 (2.4–6.0)
Lesion diameter, median (IQR), mm	25 (16–37)	25 (18–34)	0.0774	30 (20–33)	27 (20–35)
Poor Prognostic Factors					
SM3, n (%)	15 (52)	27 (51)	1	17 (45.9)	59 (50.4)
T1, n (%)	11 (38)	22 (41.5)	0.9359	21 (56.7)	54 (46.2)
T2, n (%)	11	21	1	15 (40.5)	47 (40.2)
T3, n (%)	0 (0)	1 (1.8)	NA	1 (2.7)	2 (1.7)
Poorly differentiated, n (%)	2 (6.9)	3 (5.6)	1	2 (2.7)	7 (6.0)
Lymphovascular invasion, n (%)	4 (13.8)	12 (22.6)	0.4995	11 (29.7)	27 (23.1)
Tumour budding, n (%)	4 (13.8)	4 (7.5)	0.6016	3 (8.1)	11 (9.4)
R1 resection margin, n (%)	3 (10.3)	10 (18.9)	0.4876	13 (35.1)	26 (22.2)
Adjuvant Treatment Offered					
CRT n (%)	3 (11.1)	14 (26.4)	0.4053	0	17 (14.5)
Surgery n (%)	6 (22.2)	18 (34.0)	0.0139	0	24 (20.5)
RT n (%)	11 (40.7)	14 (26.4)	0.4053	0	25 (21.4)
Surveillance n (%)	7 (26.0)	7 (13.2)	0.8671	0	14 (12.0)
Contact X-ray brachytherapy n (%)	0	0	NA	37 (100)	37 (31.6)

**Table 2:** Survival outcomes of study participants according to adjuvant treatment option.

Treatment	Follow-Up Months (Median, IQR)	Recurrence, n (%)	Time to Recurrence (Median, Months)	Deaths, n (%)	Time to Death (Median, Months)
Chemoradiotherapy	55 (21–58)	4/17 (24)	36	4/17 (24)	58
Surgery	58 (38–73)	11/24 (46)	10	7/24 (29)	44
Radiotherapy	51 (15–66)	6/25 (24)	53	8/25 (32)	55
Surveillance	34 (13–45)	4/14 (14)	7	4/14 (14)	48
CXB boost	62 (23–77)	5/37 (14)	35	25/37 (68)	66
Overall	57 (39–63)	30/117 (36)	18	48/117 (41)	61

**Table 3:** Comparison of baseline difference between CRT, TME, and contact boost arm.

Variables	CXB n (%)	CRT n (%)	TME n (%)	Level of Significance (*p* Value)			
				Overall	CXB and TME	CRT and TME	CXB and CRT
Total sample	37	17	24				
Age (median, IQR)	80 (72–84)	67 (63–76)	71.5 (67–76)	0.0991	0.0011 *	0.5823 *	0.0011 *
Tumour Stage at Diagnosis (T2 and above)							
Yes	16 (43.2)	6 (35.30)	16 (66.7)				
No	21 (56.8)	11 (64.7)	8 (33.3)	0.0923	0.1267	0.0956	0.7995
Tumour Size at Diagnosis (≤3 cm)							
Yes	25 (67.6)	11 (64.70)	16 (66.7)				
No	12 (32.4)	6 (35.3)	8 (33.3)	0.9788	1	1	1
Lympho-vascular Invasion							
Yes	11 (29.7)	2 (11.80)	8 (33.3)				
No	26 (70.3)	15 (88.2)	16 (67.7)	0.2564	0.9889	0.2243	0.275
R1 Resection Margin							
Yes	13 (35.1)	4 (23.50)	7 (29.2)				
No	24 (64.9)	13 (76.5)	17 (71.8)	0.6996	0.8368	0.9652	0.591

* Mann–Whitney U test.

## Data Availability

The raw data supporting the reported results of this study will be made available upon reasonable request. Interested researchers may contact the corresponding author, Dr. Muneeb Ul Haq, at muneeb.haq@liverpool.ac.uk, for access to the datasets. Please note that data sharing will comply with the ethical and privacy guidelines, and requests may require approval from the institutional review boards of the Royal Liverpool Hospital and Clatterbridge Cancer Center.

## Abbreviations

The following abbreviations are used in this manuscript:

CRC	colorectal cancer
ERC	early rectal cancer
TME	total mesorectal excision
TEMS	transanal endoscopic microsurgery
EUS	endorectal ultrasound
MRI	magnetic resonance imaging
CT	computed tomography
RT	radiotherapy
SM	submucosal invasion grade
CRT	chemoradiotherapy
CXB	contact X-ray brachytherapy
CRM	circumferential resection margin
RM	resection margin
MERCURY	Magnetic Resonance Imaging and Rectal Cancer European Equivalence Study criteria
SMD	standardised mean difference
PSM	propensity score matching
IPTW	inverse probability of treatment weighting
DFS	disease-free survival
OS	overall survival
HR	hazard ratio
CI	confidence interval
ASA	American Society of Anesthesiologists
BMI	body mass index
MDT	multidisciplinary team
SERC-MDT	Specialist Early Rectal Cancer Multidisciplinary Team
PET	positron emission tomography
FDG	fluorodeoxyglucose
APR	abdominoperineal resection
